# Proceedings of the First International Conference on Toxicogenomics Integrated with Environmental Sciences (TIES-2007)

**DOI:** 10.1186/1753-6561-3-s2-s1

**Published:** 2009-03-10

**Authors:** Pierre R Bushel, Dahlia Nielsen, Weida Tong

**Affiliations:** 1 Biostatistics Branch, National Institute of Environmental Health Sciences, Research Triangle Park, North Carolina, USA; 2 Department of Statistics, North Carolina State University, Raleigh, North Carolina, USA; 3 Bioinformatics Research Center, North Carolina State University, Raleigh, North Carolina, USA; 4 Center for Toxicoinformatics, National Center for Toxicological Research, Jefferson, Arkansas, USA

## Abstract

The First International Conference on Toxicogenomics Integrated with Environmental Sciences (TIES-2007) was held at the North Carolina State University McKimmon Center in Raleigh, North Carolina on October 25th and 26th, 2007. Based on the presentations at the conference and the commitment or interest of the presenters to contribute a manuscript of their research, we compiled this collection of articles as proceedings of the conference and an in-depth topical review of the utility of bioinformatics in the fields of toxicogenomics and environmental genomics.

## Overview of the conference

The First International Conference on Toxicogenomics Integrated with Environmental Sciences (TIES-2007) was held at the North Carolina State University McKimmon Center in Raleigh, North Carolina on October 25^th ^and 26^th^, 2007 and had over 120 attendees from the United States, China, Germany and Korea [1]. The TIES conferences emphasize the application of bioinformatics in the fields of toxicogenomics and environmental genomics and provide a venue where diverse scientists can exchange the current advances in bioinformatics for elucidating perturbed biological mechanisms and pathways through 'omics and advanced technologies. The theme of the TIES-2007 conference was "bridging the toxicogenomics and environmental genomics communities through bioinformatics". The conference was organized by the National Institutes of Health (NIH) – National Institute of Environmental Health Science (NIEHS), the United States Food and Drug Administration (FDA) – National Center for Toxicological Research (NCTR) and the North Carolina State University (NCSU) Bioinformatics Research Center (BRC). Rosetta Biosoftware and JMP Genomics were sponsors of the conference. TIES-2007 featured several keynote speakers, a host of invited presentations and a poster session covering topics related to toxicogenomics, proteomics, bioinformatics, epigenetics, environmental genomics and genetics, biomarker discovery, gene regulatory networks and systems biology. In addition, a special session on the FDA-led MicroArray Quality Control project [2,3] was provided that highlighted reproducibility of microarray analysis. Finally, four students competed for cash awards for the oral presentation of their poster. First place went to ClarLynda Williams of the Bioinformatics Program at the NCSU BRC, second place to Yunjung Kim of the Bioinformatics Program at the NCSU BRC, third place to Venus Welch at the Integrated Biosciences Program at Tuskegee University and fourth place to D. Ryan Georgianna at the Functional Genomics Program at the NCSU BRC.

The advent of toxicogenomics, a combination of the fields of toxicology and genomics, promises to facilitate the identification of potential human and environmental toxicants, and their putative mechanism(s) of action, through the use of genomics [4]. However, although a wealth of data has been collected and analyzed to assess risk and human health issues from exposure to toxicants, very little is still known about the biological processes that account for idiosyncratic toxicity or human genetics susceptibility to environmental stressors. Fortunately, environmental health sciences and genomics have taken form to leverage environmental exposures for a better understanding of the role of gene and gene-product expression and genetic variation in the development and progression of complex human diseases [5]. One of the main challenges in toxicogenomics and environmental genomics is managing and making sense of the abundance of the data to elucidate the interaction between genes and the environment in the development and progression of human diseases [6-10]. The papers presented in the TIES-2007 proceedings provide a glimpse of the utility of bioinformatics in the fields of toxicogenomics and environmental genomics, and a peak at the research advances and technologic developments at the forefront of these disciplines. Below is a synopsis of the student oral presentations and the MAQC special session that took place at TIES-2007.

### Student oral presentations

ClarLynda Williams, a doctoral candidate in the bioinformatics program at NCSU, Maritja Wolf from Lockheed Martin and Ann Richards, her mentor at the Environmental Protection Agency (EPA), aimed to chemically index the content of public genomic databases to make the data accessible in relation to other publicly available, chemically-indexed toxicological information. The five public genomic databases that contained data of chemogenomic interest were the Chemical Effects in Biological Systems (CEBS) knowledgebase, Public Expression Profiling Resources (PEPR) web database, ArrayExpress genomic repository, the Gene Expression Omnibus (GEO) repository, and the Environment, Drugs, and Gene Expression database (EDGE). After chemical exposure experiments were identified, the chemical space was defined through structural similarity and compared to the chemical space of public toxicological data from the EPA DSSTox project [11]. By evaluating the chemical space of public genomic data in relation to public toxicological data, it was possible to identify classes of chemicals on which to develop methodologies for the integration of chemogenomic data into predictive toxicology by comparisons of experimental data across labs, chemicals, platforms and species.

Yunjung Kim, a doctoral candidate in the bioinformatics program at NCSU and Zhao-Bang Zeng, her mentor at the NCSU BRC, aimed to understand the multilocus linkage disequlibrium (LD) structure among SNPs in the human genome by testing hypotheses about 2- and 3-locus gametic disequilibrium via a resampling method. The usual way to test hypotheses about different orders of gametic disequilibrium is the likelihood ratio test (LRT). With large samples, the distribution of LRT statistic approximates a chi-square distribution with the degree of freedom equal to the difference of parameter numbers between the null model and the alternative model. For some tests such as two-locus gametic disequilibrium, the chi-square approximation still works very well. However, for tests of 3-locus or more gametic disequilibria, the asymptotic chi-square approximation no longer works especially when there are many unobserved haplotypes. As an alternative choice, they used a resampling method suggested by Long *et al. *[12] and constructed empirical distributions of statistics by resampling the observed data. Such empirical distributions avoid the large sample assumption at the expense of more computing time and may provide more reliable p-values for the test of higher order LD. This resampling method is illustrated with simulation experiments and analysis of the phased haplotype data from DeLuca *et al. *[13].

Venus Welch, a graduate student in integrative biosciences with a focus in environmental toxicology and toxicogenomics at Tuskegee University and Pierre R. Bushel, her mentor at NIEHS, embarked on a summer internship project in the Microarray and Genome Informatics group at NIEHS to ascertain the differences in gene expression patterns and biological pathways in the livers of rats exposed to chemical agents. Microarray gene expression data acquired from the livers of male Fisher rats, orally dosed with 1, 2- or 1, 4-dichlorobenzene (DCB, isomers used in pesticides) in single doses of 15, 150, and 1500 mg/kg for 6 and 24 hours was analyzed. A set of 463 genes that are involved in toxic response pathways were used to perform clustering and principal component analysis of the data resulting in a separation of the exposed animals by the toxic dose in 3-D space. Interestingly, at the 150 mg/kg for 24-hour exposure, ALT and AST enzyme levels from the 1, 4-DCB-treated animals were equivalent to 1, 2-DCB-control animals, where no necrosis was observed. However, the 1, 2-DCB-treated animals revealed elevated levels of the enzymes as well as necrosis in comparison to control and 1, 4-DCB-treated animals. These end-point measurements were effective in anchoring the gene expression to the phenotype of the samples for pathways analysis. A central regulating role of tumor necrosis factor and genes that influence, or are related to, apoptosis, MAP kinase signaling and metabolism in the liver were revealed. However, *carnitine palmitoyltransferase 2 *was found to be differentially expressed between the pathways generated from the 1, 2- and the 1, 4-DCB 150 mg/kg for 24 hours treated animals. Recent evidence from the gene expression analysis of a compendium of hepatotoxicants (including this 1,2- and 1,4-DCB data) supports these findings [14].

D. Ryan Georgianna, a doctoral candidate in the functional genomics program at NCSU, his mentor Gary A. Payne and others in the Department of Plant Pathology at NCSU, utilized an adaptation of the stable isotope labelling by amino acids on cell culture (SILAC) procedure to reliably quantify alterations in protein concentrations in response to temperature changes that regulate the biosynthesis of aflatoxin (a carcinogenic mycotoxin produced by *Aspergillus flavus *– a fungus found on several commodities such as corn, peanuts, cotton and tree nuts) [15]. SILAC relies on the quantitative incorporation of labelled amino acids into proteins to provide a powerful mass spectrometry based proteomics tool for rapid quantification of proteins. Aflatoxin production is inhibited at 37°C, the optimum temperature for growth of *A. flavus*. The comparison between conducive (28°C) and nonconducive (37°C) temperatures for aflatoxin biosynthesis revealed 31 proteins more abundant at 37°C and 18 more abundant at 28°C. Interestingly, the particular changes in the level of expression of the aflatoxin pathway enzymes seemed to closely follow the strong repression of both aflatoxin biosynthesis and transcription of the aflatoxin pathway genes observed at 37°C. Transcripts were present for 379 proteins quantified by SILAC, but their expression did not always correlate well with transcript levels of encoding genes. This is the first reported labelling of a multicellular (whole organism) free-living prototroph using the SILAC procedure to compare ^13^C_6_-arginine-labeled samples to ^12^C_6_-arginine-labeled samples for quantitative proteomics.

### MicroArray Quality Control (MAQC) special session

The MAQC project is an FDA-led, community-wide effort aimed at reaching consensus within the microarray research community on best ways to assess quality, analyze and apply DNA microarrays. The MAQC project is divided into two phases, the phase I effort (MAQC-I) is to address the technical issues related to use of microarrays while phase II (MAQC-II) is focused on the issues related to the application of microarrays in clinics and risk assessment. This special session is to communicate the MAQC-I results and conclusions with the research community through the TIES meeting.

Dr. Welda Tong from the FDA's National Center for Toxicological Research, a principle investigator in MAQC, started the session with an overview of the MAQC-I project and the status of the MAQC-II program. Specifically, he emphasized the impact of this project to the regulatory use of the microarray-based data. He indicated that the lessons learned from MAQC are paving the way for development of a Best Practice Guidance Document for future voluntary as well as regular submissions of pharmacogenomics data to the FDA and he indicated that, such a best practice document draft, a companion document to "Guidance for Industry – Pharmacogenomic Data Submission" was recently released for comments.

MAQC-I used six different commercial and one institutionally developed microarray platforms. Over 130 scientists from 51 organizations participated in the generation and analysis of this dataset, and important conclusions were drawn from this collaborative effort. Dr. Wendell Jones from Expression Analysis summarized the main findings and conclusions from MAQC-I. Specifically, when standard operating procedures for an assay are followed and the data is analyzed properly, the following is demonstrated: (1) High repeatability (within site) and reproducibility (between sites) for each platform; (2) High cross-platform comparability, including one- vs. two-color platforms; and (3) High correlation between quantitative gene expression (e.g. TaqMan) and microarray platforms, where the few discordant measurements were found, mainly, due to probe sequence and thus target location. These findings were further discussed and demonstrated in more detail by Dr. Yulin Luo's (Panomics, Inc.) presentation on "Evaluation of DNA Microarray Results with Quantitative Gene Expression Platforms", Dr. Russ Wolfinger's (SAS) presentation on "Performance Comparison of One-Color and Two-Color Platforms", and Dr. Anne Lucas' (Agilent Technologies) presentation on "Evaluation of External RNA Controls for the Assessment of Microarray Performance".

The MAQC-I evaluated various gene selection rules to determine the reproducible gene lists across labs and across platforms. It was found that within-lab, cross-lab and cross-platform reproducibility is likely to be reached if fold change (FC) is used as a primary ranking/selection criterion but not to be reached if p-value from a simple T-test is used as a primary ranking/selection criterion. Dr. Ed Lobenhofer (Cogenics, Inc.) discussed the results from the rat toxicogenomic study of this project to demonstrate the MAQC findings; i.e., FC ranking coupled with a less stringent P-value cutoff might be a reasonable way to balance reproducibility and statistical significance. These findings could be one of the most important but also most controversial conclusions from MAQC-I. Consequently, Dr. Russ Wolfinger (SAS) presented a set of new studies based on a simulated dataset to further illustrate the relationship between the reproducibility and specificity/sensitivity in microarray analysis. He demonstrated that there is no monotonic relationship between reproducibility and specificity/sensitivity and then concluded that care should be taken to align the selected analysis methods with the study objectives. It appears that more extensive studies on this subject are needed.

## Future conferences

The next TIES conference will be held in Seoul, South Korea in conjunction with a Symposium of the 5th International Conference for Toxicogenomics, September 21-23, 2009. TIES-2009 will be a joint conference with the Korea Society on Toxicogenomics and Toxicoproteomics, NIEHS, NCTR and the NCSU-BRC. The conference will be held at the Renaissance Seoul Hotel. The conference web sites will be http://www.ties-conference-2009.org and  http://www.tox.or.kr/toxsoc/event/ict-2009/.

## Competing interests

The authors declare that they have no competing interests.

## Authors' contributions

PRB wrote the majority of this introduction to the proceedings, WT contributed to the proceedings summary section and wrote the synopsis of the MAQC special session section. All authors served as co-editors for the proceedings with PRB serving as Senior Editor.
